# Poly[[di-μ_3_-nicotinato-μ_3_-oxalato-samarium(III)silver(I)] dihydrate]

**DOI:** 10.1107/S1600536809032115

**Published:** 2009-08-19

**Authors:** Li-Cai Zhu, Zhen-Gang Zhao, Shu-Juan Yu

**Affiliations:** a School of Chemistry and Environment, South China Normal University, Guangzhou 510631, People’s Republic of China; b College of Light Industry and Food Sciences, South China University of Technology, Guangzhou 510641, People’s Republic of China

## Abstract

In the title three-dimensional heterometallic complex, {[AgSm(C_6_H_4_NO_2_)_2_(C_2_O_4_)]·2H_2_O}_*n*_, the Sm^III^ ion is eight-coordinated by four O atoms from four different nicotinate ligands and four O atoms from two different oxalate ligands. The three-coordinate Ag^I^ ion is bonded to two N atoms from two different nicotinate anions and one O atom from an oxalate anion. These metal coordination units are connected by bridging nicotinate and oxalate ligands, generating a three-dimensional network. The uncoordinated water mol­ecules link the carboxyl­ate groups *via* O—H⋯O hydrogen bonding. The crystal structure is further stabilized by hydrogen bonds between the water mol­ecules.

## Related literature

For theapplications of lanthanide–transition metal heterometallic complexes with bridging multifunctional organic ligands, see: Cheng *et al.* (2006 ▶); Kuang *et al.* (2007 ▶); Luo *et al.* (2007 ▶); Peng *et al.* (2008 ▶).
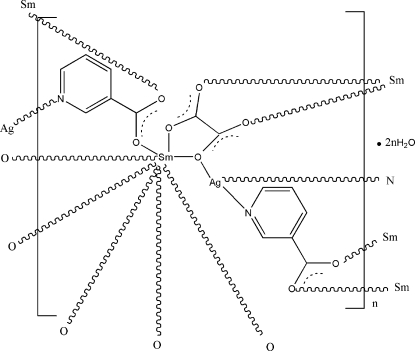


## Experimental

### 

#### Crystal data

[AgSm(C_6_H_4_NO_2_)_2_(C_2_O_4_)]·2H_2_O
*M* *_r_* = 626.49
Monoclinic, 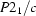

*a* = 9.7145 (9) Å
*b* = 22.3444 (15) Å
*c* = 9.1726 (6) Å
β = 117.295 (1)°
*V* = 1769.4 (2) Å^3^
*Z* = 4
Mo *K*α radiation
μ = 4.45 mm^−1^
*T* = 296 K
0.23 × 0.20 × 0.19 mm

#### Data collection

Bruker APEXII area-detector diffractometer
Absorption correction: multi-scan (*SADABS*; Sheldrick, 1996 ▶) *T*_min_ = 0.374, *T*_max_ = 0.429
8972 measured reflections
3171 independent reflections
2995 reflections with *I* > 2σ(*I*)
*R*_int_ = 0.027

#### Refinement

*R*[*F* ^2^ > 2σ(*F* ^2^)] = 0.023
*wR*(*F* ^2^) = 0.052
*S* = 1.12
3171 reflections
254 parameters
H-atom parameters constrained
Δρ_max_ = 0.84 e Å^−3^
Δρ_min_ = −0.65 e Å^−3^

### 

Data collection: *APEX2* (Bruker, 2004 ▶); cell refinement: *SAINT* (Bruker, 2004 ▶); data reduction: *SAINT*; program(s) used to solve structure: *SHELXS97* (Sheldrick, 2008 ▶); program(s) used to refine structure: *SHELXL97* (Sheldrick, 2008 ▶); molecular graphics: *SHELXTL* (Sheldrick, 2008 ▶); software used to prepare material for publication: *SHELXL97*.

## Supplementary Material

Crystal structure: contains datablocks I, global. DOI: 10.1107/S1600536809032115/pv2185sup1.cif

Structure factors: contains datablocks I. DOI: 10.1107/S1600536809032115/pv2185Isup2.hkl

Additional supplementary materials:  crystallographic information; 3D view; checkCIF report

## Figures and Tables

**Table 1:** Hydrogen-bond geometry (Å, °)

*D*—H⋯*A*	*D*—H	H⋯*A*	*D*⋯*A*	*D*—H⋯*A*
O1*W*—H1*W*⋯O7^i^	0.86	2.10	2.960 (5)	175
O1*W*—H2*W*⋯O2*W*	0.86	2.06	2.892 (7)	161
O2*W*—H4*W*⋯O1*W*^i^	0.87	1.92	2.780 (7)	171

Symmetry code: (i) 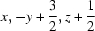.

**Fig. 1.**
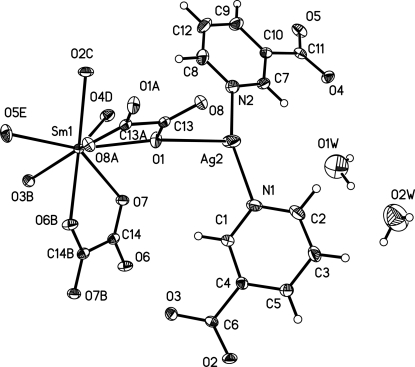
The molecular structure showing the atomic-numbering scheme and displacement ellipsoids drawn at the 30% probability level. Symmetry codes included in the atomic labels: (A) 2-x, 2-y, 1-z; (B) 1-x, 2-y, -z; (C) 1+x, y, z; (D) x, 1.5-y, -0.5+z; (E) 2-x, 0.5+y, 0.5-z.

**Fig. 2.**
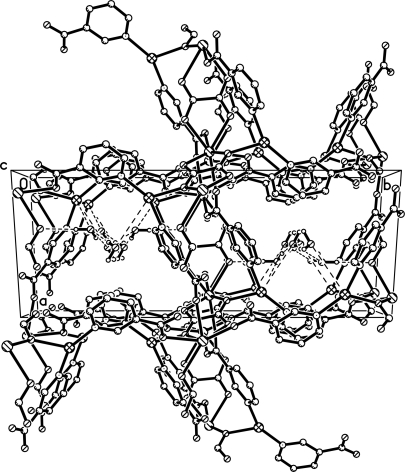
A view of the three-dimensional structure of the title compound; dotted lines denote hydrogen bonds.

## supplementary crystallographic information

### Comment

In the past few years, lanthanide-transition metal heterometallic complexs with bridging multifunctionnal organic ligands have generated much interest, not only because of their impressive topological structures, but also due to their versatile applications in ion exchange, magnetism, bimetallic catalysis and luminescent probe (Cheng *et al.*, 2006; Kuang *et al.*, 2007; Luo *et al.*, 2007; Peng *et al.*, 2008). As an extension of this research, we report here the structure of the title compound, a new heterometallic coordination polymer.

In the title compound (Fig. 1), there are one Sm^III^ ion, one Ag^I^ ion, two halves of oxalate ligand, two nicotinate ligands, and two lattice water molecules in the asymmetric unit. Each Sm^III^ ion is eight-coordinated by four O atoms from four different nicotinate ligands, and four O atoms of two different oxalate ligands. The Sm center can be described as having a bicapped trigonal prism coordination geometry. The three-coordinate Ag^I^ ion is bonded to two N atoms from two different nicotinate anions and one O atom from an oxalate anion. Thus the Ag^I^ ion is in a T-shaped configuration. These metal coordination units are connected by bridging nicotinate and oxalate ligands, generating a three-dimensional network (Fig. 2). The uncoordinated water molecules link the carboxylate groups by O—H···O hydrogen bonding (Table 1). The crystal structure is further stabilized by hydrogen bonds.

### Experimental

A mixture of AgNO_3_ (0.057 g, 0.33 mmol), Sm_2_O_3_ (0.116 g, 0.33 mmol), nicotinic acid (0.164 g, 1.33 mmol), oxalic acid (0.119 g, 1.33 mmol), H_2_O (7 ml), and HClO_4_ (0.257 mmol)(pH 2) was sealed in a 20 ml Teflon-lined reaction vessel at 443 K for 6 days then slowly cooled to room temperature. The product was collected by filtration, washed with water and air-dried. Colorless block crystals suitable for X-ray analysis were obtained.

### Refinement

H atoms bonded to C atoms were positioned geometrically and refined as riding, with C—H = 0.93 Å and *U*_iso_(H) = 1.2 *U*_eq_(C). H atoms of water molecules were found from difference Fourier maps and included in the refinements with a restraint of O—H = 0.86 - 0.87 Å and *U*_iso_(H) = 1.5 *U*_eq_(O). The largest residual electron density in the final difference map was located at a distance of 0.82 Å from Ag2 atom and was meaningless.

### Crystal data

**Table:**

[AgSm(C_6_H_4_NO_2_)_2_(C_2_O_4_)]·2H_2_O	*F*(000) = 1196
*M**_r_* = 626.49	*D*_x_ = 2.352 Mg m^−^^3^
Monoclinic, *P*2_1_/*c*	Mo *K*α radiation, λ = 0.71073 Å
Hall symbol: -P 2ybc	Cell parameters from 6346 reflections
*a* = 9.7145 (9) Å	θ = 2.4–27.8°
*b* = 22.3444 (15) Å	µ = 4.45 mm^−^^1^
*c* = 9.1726 (6) Å	*T* = 296 K
β = 117.295 (1)°	Block, colorless
*V* = 1769.4 (2) Å^3^	0.23 × 0.20 × 0.19 mm
*Z* = 4	

### Data collection

**Table:**

Bruker APEXII area-detector diffractometer	3171 independent reflections
Radiation source: fine-focus sealed tube	2995 reflections with *I* > 2σ(*I*)
graphite	*R*_int_ = 0.027
φ and ω scans	θ_max_ = 25.2°, θ_min_ = 2.4°
Absorption correction: multi-scan (*SADABS*; Sheldrick, 1996)	*h* = −5→11
*T*_min_ = 0.374, *T*_max_ = 0.429	*k* = −26→26
8972 measured reflections	*l* = −10→10

### Refinement

**Table:**

Refinement on *F*^2^	Secondary atom site location: difference Fourier map
Least-squares matrix: full	Hydrogen site location: inferred from neighbouring sites
*R*[*F*^2^ > 2σ(*F*^2^)] = 0.023	H-atom parameters constrained
*wR*(*F*^2^) = 0.052	*w* = 1/[σ^2^(*F*_o_^2^) + (0.0174*P*)^2^ + 1.7329*P*] where *P* = (*F*_o_^2^ + 2*F*_c_^2^)/3
*S* = 1.12	(Δ/σ)_max_ = 0.002
3171 reflections	Δρ_max_ = 0.84 e Å^−^^3^
254 parameters	Δρ_min_ = −0.65 e Å^−^^3^
0 restraints	Extinction correction: *SHELXL97* (Sheldrick, 2008), Fc^*^=kFc[1+0.001xFc^2^λ^3^/sin(2θ)]^-1/4^
Primary atom site location: structure-invariant direct methods	Extinction coefficient: 0.00351 (16)

### Special details

**Table:**

Geometry. All e.s.d.'s (except the e.s.d. in the dihedral angle between two l.s. planes) are estimated using the full covariance matrix. The cell e.s.d.'s are taken into account individually in the estimation of e.s.d.'s in distances, angles and torsion angles; correlations between e.s.d.'s in cell parameters are only used when they are defined by crystal symmetry. An approximate (isotropic) treatment of cell e.s.d.'s is used for estimating e.s.d.'s involving l.s. planes.
Refinement. Refinement of *F*^2^ against ALL reflections. The weighted *R*-factor *wR* and goodness of fit *S* are based on *F*^2^, conventional *R*-factors *R* are based on *F*, with *F* set to zero for negative *F*^2^. The threshold expression of *F*^2^ > σ(*F*^2^) is used only for calculating *R*-factors(gt) *etc*. and is not relevant to the choice of reflections for refinement. *R*-factors based on *F*^2^ are statistically about twice as large as those based on *F*, and *R*- factors based on ALL data will be even larger.

### Fractional atomic coordinates and isotropic or equivalent isotropic displacement parameters (Å^2^)

**Table:**

	*x*	*y*	*z*	*U*_iso_*/*U*_eq_	
Sm1	0.85715 (2)	0.991315 (8)	0.11767 (2)	0.01683 (8)	
Ag2	0.82067 (4)	0.852371 (14)	0.48309 (5)	0.04794 (12)	
O1	0.8835 (3)	0.94402 (11)	0.3695 (3)	0.0275 (6)	
N1	0.6254 (4)	0.88411 (15)	0.5163 (4)	0.0346 (8)	
C4	0.3834 (4)	0.93617 (16)	0.4147 (4)	0.0231 (8)	
C3	0.4789 (5)	0.88129 (19)	0.6648 (5)	0.0368 (10)	
H3	0.4696	0.8681	0.7560	0.044*	
C1	0.5154 (4)	0.91951 (17)	0.4047 (5)	0.0291 (9)	
H1	0.5291	0.9334	0.3166	0.035*	
C2	0.6042 (5)	0.86566 (19)	0.6441 (5)	0.0382 (10)	
H2	0.6788	0.8410	0.7221	0.046*	
C6	0.2586 (4)	0.97226 (16)	0.2811 (4)	0.0222 (8)	
C5	0.3662 (5)	0.91690 (17)	0.5490 (4)	0.0293 (9)	
H5	0.2795	0.9279	0.5608	0.035*	
O3	0.2900 (3)	0.99674 (11)	0.1769 (3)	0.0284 (6)	
O2	0.1309 (3)	0.97383 (13)	0.2834 (3)	0.0313 (6)	
N2	0.9619 (4)	0.78296 (14)	0.4537 (4)	0.0332 (8)	
C7	0.9364 (4)	0.72618 (16)	0.4792 (5)	0.0287 (8)	
H7	0.8605	0.7184	0.5117	0.034*	
C9	1.1309 (5)	0.6899 (2)	0.4136 (5)	0.0390 (10)	
H9	1.1876	0.6589	0.3994	0.047*	
C8	1.0722 (5)	0.79305 (19)	0.4069 (5)	0.0408 (10)	
H8	1.0903	0.8322	0.3859	0.049*	
O7	0.6156 (3)	0.93618 (11)	0.0496 (3)	0.0253 (6)	
C10	1.0169 (4)	0.67816 (16)	0.4599 (4)	0.0239 (8)	
C12	1.1588 (5)	0.7483 (2)	0.3889 (6)	0.0490 (12)	
H12	1.2365	0.7573	0.3600	0.059*	
C13	0.9793 (4)	0.96697 (16)	0.5028 (4)	0.0207 (7)	
O8	1.0418 (3)	0.94114 (11)	0.6386 (3)	0.0255 (6)	
O6	0.3580 (3)	0.94430 (11)	−0.0787 (3)	0.0280 (6)	
O1W	0.6113 (6)	0.69616 (19)	0.5607 (5)	0.0925 (14)	
H1W	0.6115	0.6576	0.5630	0.139*	
H2W	0.5551	0.7081	0.6053	0.139*	
O2W	0.4884 (7)	0.7331 (2)	0.7801 (6)	0.1179 (19)	
H3W	0.4044	0.7508	0.7119	0.177*	
H4W	0.5226	0.7525	0.8720	0.177*	
C11	0.9771 (4)	0.61643 (15)	0.4922 (4)	0.0236 (8)	
O4	0.8882 (3)	0.61157 (11)	0.5557 (3)	0.0322 (6)	
O5	1.0350 (3)	0.57246 (12)	0.4552 (3)	0.0353 (7)	
C14	0.4919 (4)	0.96531 (16)	−0.0083 (4)	0.0216 (8)	

### Atomic displacement parameters (Å^2^)

**Table:**

	*U*^11^	*U*^22^	*U*^33^	*U*^12^	*U*^13^	*U*^23^
Sm1	0.01410 (11)	0.01776 (12)	0.01771 (11)	0.00069 (7)	0.00649 (8)	0.00163 (7)
Ag2	0.0358 (2)	0.02335 (18)	0.0903 (3)	0.00795 (13)	0.0338 (2)	0.00929 (16)
O1	0.0327 (16)	0.0290 (14)	0.0187 (12)	−0.0129 (12)	0.0100 (12)	−0.0031 (10)
N1	0.0243 (18)	0.0306 (19)	0.046 (2)	0.0061 (14)	0.0134 (16)	0.0060 (15)
C4	0.021 (2)	0.026 (2)	0.0181 (17)	−0.0007 (15)	0.0052 (15)	0.0010 (14)
C3	0.044 (3)	0.042 (3)	0.024 (2)	0.010 (2)	0.0148 (19)	0.0092 (17)
C1	0.023 (2)	0.032 (2)	0.032 (2)	0.0064 (16)	0.0126 (17)	0.0080 (17)
C2	0.032 (3)	0.037 (2)	0.034 (2)	0.0069 (19)	0.005 (2)	0.0100 (18)
C6	0.0167 (19)	0.0244 (19)	0.0190 (17)	0.0007 (15)	0.0027 (15)	−0.0008 (14)
C5	0.026 (2)	0.035 (2)	0.027 (2)	0.0038 (17)	0.0124 (17)	0.0024 (17)
O3	0.0231 (15)	0.0376 (16)	0.0205 (13)	0.0005 (11)	0.0066 (11)	0.0056 (11)
O2	0.0161 (14)	0.0495 (17)	0.0254 (14)	0.0082 (12)	0.0070 (11)	0.0042 (12)
N2	0.0314 (19)	0.0210 (17)	0.048 (2)	0.0001 (14)	0.0188 (16)	0.0057 (15)
C7	0.024 (2)	0.023 (2)	0.041 (2)	0.0007 (16)	0.0171 (18)	0.0021 (16)
C9	0.036 (3)	0.037 (3)	0.053 (3)	0.0067 (19)	0.029 (2)	0.005 (2)
C8	0.044 (3)	0.031 (2)	0.053 (3)	−0.0032 (19)	0.026 (2)	0.010 (2)
O7	0.0179 (14)	0.0232 (13)	0.0326 (14)	0.0020 (11)	0.0097 (11)	0.0017 (11)
C10	0.022 (2)	0.0234 (19)	0.0251 (18)	0.0017 (15)	0.0098 (16)	−0.0018 (15)
C12	0.041 (3)	0.051 (3)	0.071 (3)	−0.002 (2)	0.040 (3)	0.011 (2)
C13	0.0181 (19)	0.0234 (19)	0.0239 (19)	0.0007 (14)	0.0126 (16)	0.0006 (14)
O8	0.0291 (15)	0.0218 (13)	0.0221 (13)	0.0012 (11)	0.0088 (11)	0.0022 (10)
O6	0.0179 (14)	0.0271 (14)	0.0371 (15)	−0.0011 (11)	0.0109 (12)	−0.0066 (11)
O1W	0.120 (4)	0.060 (3)	0.108 (3)	0.008 (3)	0.062 (3)	−0.012 (2)
O2W	0.147 (5)	0.100 (4)	0.106 (4)	0.027 (4)	0.056 (4)	0.012 (3)
C11	0.024 (2)	0.0162 (18)	0.0233 (18)	0.0018 (14)	0.0045 (16)	−0.0033 (14)
O4	0.0308 (16)	0.0235 (14)	0.0488 (17)	−0.0014 (11)	0.0239 (14)	0.0039 (12)
O5	0.0417 (18)	0.0265 (15)	0.0296 (14)	0.0111 (12)	0.0092 (13)	−0.0050 (11)
C14	0.021 (2)	0.025 (2)	0.0208 (18)	−0.0003 (15)	0.0115 (16)	−0.0035 (14)

### Geometric parameters (Å, °)

**Table:**

Sm1—O5^i^	2.340 (3)	N2—C7	1.334 (5)
Sm1—O2^ii^	2.414 (2)	N2—C8	1.342 (5)
Sm1—O4^iii^	2.420 (3)	C7—C10	1.386 (5)
Sm1—O3^iv^	2.424 (2)	C7—H7	0.9300
Sm1—O6^iv^	2.425 (2)	C9—C12	1.372 (6)
Sm1—O1	2.444 (2)	C9—C10	1.381 (5)
Sm1—O7	2.464 (2)	C9—H9	0.9300
Sm1—O8^v^	2.496 (2)	C8—C12	1.366 (6)
Ag2—N2	2.168 (3)	C8—H8	0.9300
Ag2—N1	2.174 (3)	O7—C14	1.251 (4)
Ag2—O1	2.497 (2)	C10—C11	1.498 (5)
O1—C13	1.257 (4)	C12—H12	0.9300
N1—C2	1.344 (5)	C13—O8	1.249 (4)
N1—C1	1.346 (5)	C13—C13^v^	1.537 (7)
C4—C1	1.378 (5)	O8—Sm1^v^	2.496 (2)
C4—C5	1.385 (5)	O6—C14	1.249 (4)
C4—C6	1.504 (5)	O6—Sm1^iv^	2.425 (2)
C3—C2	1.361 (6)	O1W—H1W	0.8624
C3—C5	1.376 (5)	O1W—H2W	0.8612
C3—H3	0.9300	O2W—H3W	0.8629
C1—H1	0.9300	O2W—H4W	0.8667
C2—H2	0.9300	C11—O4	1.249 (4)
C6—O2	1.251 (4)	C11—O5	1.253 (4)
C6—O3	1.254 (4)	O4—Sm1^vii^	2.420 (3)
C5—H5	0.9300	O5—Sm1^viii^	2.340 (3)
O3—Sm1^iv^	2.424 (2)	C14—C14^iv^	1.559 (7)
O2—Sm1^vi^	2.414 (2)		
			
O5^i^—Sm1—O2^ii^	78.29 (10)	N1—C2—C3	123.2 (4)
O5^i^—Sm1—O4^iii^	123.28 (10)	N1—C2—H2	118.4
O2^ii^—Sm1—O4^iii^	76.96 (9)	C3—C2—H2	118.4
O5^i^—Sm1—O3^iv^	73.05 (9)	O2—C6—O3	126.2 (3)
O2^ii^—Sm1—O3^iv^	129.50 (9)	O2—C6—C4	115.9 (3)
O4^iii^—Sm1—O3^iv^	85.19 (9)	O3—C6—C4	117.9 (3)
O5^i^—Sm1—O6^iv^	88.09 (10)	C3—C5—C4	119.2 (4)
O2^ii^—Sm1—O6^iv^	144.58 (9)	C3—C5—H5	120.4
O4^iii^—Sm1—O6^iv^	136.12 (9)	C4—C5—H5	120.4
O3^iv^—Sm1—O6^iv^	75.08 (9)	C6—O3—Sm1^iv^	132.3 (2)
O5^i^—Sm1—O1	137.60 (8)	C6—O2—Sm1^vi^	143.9 (2)
O2^ii^—Sm1—O1	74.16 (9)	C7—N2—C8	117.1 (4)
O4^iii^—Sm1—O1	80.84 (9)	C7—N2—Ag2	118.6 (3)
O3^iv^—Sm1—O1	148.63 (9)	C8—N2—Ag2	124.2 (3)
O6^iv^—Sm1—O1	96.06 (9)	N2—C7—C10	123.6 (4)
O5^i^—Sm1—O7	144.52 (9)	N2—C7—H7	118.2
O2^ii^—Sm1—O7	136.44 (9)	C10—C7—H7	118.2
O4^iii^—Sm1—O7	70.86 (9)	C12—C9—C10	118.5 (4)
O3^iv^—Sm1—O7	76.49 (9)	C12—C9—H9	120.7
O6^iv^—Sm1—O7	66.61 (8)	C10—C9—H9	120.7
O1—Sm1—O7	72.42 (8)	N2—C8—C12	122.8 (4)
O5^i^—Sm1—O8^v^	75.15 (9)	N2—C8—H8	118.6
O2^ii^—Sm1—O8^v^	70.51 (9)	C12—C8—H8	118.6
O4^iii^—Sm1—O8^v^	138.13 (9)	C14—O7—Sm1	117.7 (2)
O3^iv^—Sm1—O8^v^	136.13 (8)	C9—C10—C7	118.1 (4)
O6^iv^—Sm1—O8^v^	74.44 (8)	C9—C10—C11	123.5 (3)
O1—Sm1—O8^v^	65.62 (8)	C7—C10—C11	118.4 (3)
O7—Sm1—O8^v^	117.85 (8)	C8—C12—C9	119.8 (4)
N2—Ag2—N1	153.35 (12)	C8—C12—H12	120.1
N2—Ag2—O1	104.18 (11)	C9—C12—H12	120.1
N1—Ag2—O1	100.77 (11)	O8—C13—O1	125.9 (3)
C13—O1—Sm1	116.9 (2)	O8—C13—C13^v^	117.5 (4)
C13—O1—Ag2	98.2 (2)	O1—C13—C13^v^	116.6 (4)
Sm1—O1—Ag2	144.02 (10)	C13—O8—Sm1^v^	115.2 (2)
C2—N1—C1	117.2 (4)	C14—O6—Sm1^iv^	118.5 (2)
C2—N1—Ag2	120.9 (3)	H1W—O1W—H2W	106.9
C1—N1—Ag2	121.7 (3)	H3W—O2W—H4W	106.8
C1—C4—C5	118.1 (3)	O4—C11—O5	123.4 (3)
C1—C4—C6	121.2 (3)	O4—C11—C10	118.0 (3)
C5—C4—C6	120.7 (3)	O5—C11—C10	118.7 (3)
C2—C3—C5	119.1 (4)	C11—O4—Sm1^vii^	112.3 (2)
C2—C3—H3	120.5	C11—O5—Sm1^viii^	179.0 (3)
C5—C3—H3	120.5	O6—C14—O7	126.5 (3)
N1—C1—C4	123.1 (4)	O6—C14—C14^iv^	117.3 (4)
N1—C1—H1	118.4	O7—C14—C14^iv^	116.2 (4)
C4—C1—H1	118.4		

Symmetry codes: (i) −*x*+2, *y*+1/2, −*z*+1/2; (ii) *x*+1, *y*, *z*; (iii) *x*, −*y*+3/2, *z*−1/2; (iv) −*x*+1, −*y*+2, −*z*; (v) −*x*+2, −*y*+2, −*z*+1; (vi) *x*−1, *y*, *z*; (vii) *x*, −*y*+3/2, *z*+1/2; (viii) −*x*+2, *y*−1/2, −*z*+1/2.

### Hydrogen-bond geometry (Å, °)

**Table:**

*D*—H···*A*	*D*—H	H···*A*	*D*···*A*	*D*—H···*A*
O1W—H1W···O7^vii^	0.86	2.10	2.960 (5)	175
O1W—H2W···O2W	0.86	2.06	2.892 (7)	161
O2W—H4W···O1W^vii^	0.87	1.92	2.780 (7)	171

Symmetry codes: (vii) *x*, −*y*+3/2, *z*+1/2.
