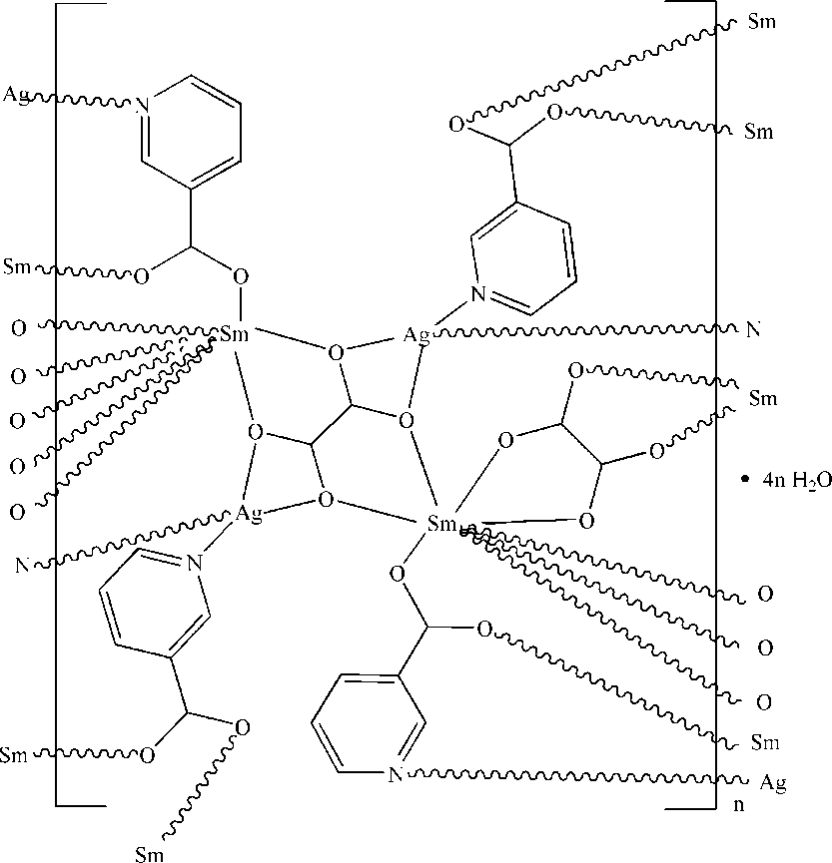# Poly[[di-μ_3_-nicotinato-μ_3_-oxalato-samarium(III)silver(I)] dihydrate]. Corrigendum

**DOI:** 10.1107/S1600536810002539

**Published:** 2010-01-30

**Authors:** Li-Cai Zhu, Zhen-Gang Zhao, Shu-Juan Yu

**Affiliations:** a School of Chemistry and Environment, South China Normal University, Guangzhou 510631, People’s Republic of China; b College of Light Industry and Food Sciences, South China University of Technology, Guangzhou 510641, People’s Republic of China

## Abstract

The title of the paper by Zhu, Zhao & Yu [*Acta Cryst.* (2009), E**65**, m1105] is corrected.

## Abstract 2

Corrigendum to *Acta Cryst.* (2009), E**65**, m1105.

In the paper by Zhu *et al.* (2009) ▶, the chemical name given in the *Title* should be ‘Poly[[tetra-μ_3_-nicotinato-μ_4_-oxalato-μ_2_-oxalato-disamarium(III)disilver(I)] tetrahydrate]’. The revised scheme is shown below.